# Faecal immunochemical test to triage patients with possible colorectal cancer symptoms: insights from the New Zealand FIT for symptomatic pilot

**DOI:** 10.3389/fgstr.2025.1622258

**Published:** 2025-09-22

**Authors:** Kai Sheng Saw, Lu-ana Ngatai, Cathy Whiteside, Susan Parry, Ian Bissett

**Affiliations:** ^1^ Department of Surgery, Faculty of Medical and Health Sciences, The University of Auckland, Auckland, New Zealand; ^2^ Screening and Health Services, Health New Zealand Waikato District, Hamilton, New Zealand; ^3^ National Bowel Screening Programme, National Public Health Service – Health, Wellington, New Zealand; ^4^ Department of Medicine, Faculty of Medical and Health Sciences, The University of Auckland, Auckland, New Zealand

**Keywords:** colorectal neoplasms/diagnosis, occult blood, triage/methods, faeces/chemistry, haemoglobins/analysis, immunochemistry

## Abstract

**Background:**

The New Zealand (NZ) FIT for Symptomatic Pilot (FSP) aimed to determine the feasibility of using faecal immunochemical test (FIT) as a triaging tool to assess patients presenting with symptoms suspicious for colorectal cancer (CRC).

**Methods:**

This is a double-blinded diagnostic accuracy study conducted in two Health NZ Districts from July 2022 to January 2024. Consecutive adult patients referred with symptoms of suspected CRC, who were triaged for colonoscopy by endoscopists, were invited to perform a quantitative FIT. The diagnostic performance of FIT for CRC was assessed.

**Results:**

Valid FIT results were returned by 1,158 (82%) of 1,413 eligible patients; 1,043 were included in the diagnostic accuracy analysis. At low (“rule-out”) faecal haemoglobin (f-Hb) thresholds, the sensitivity and specificity for CRC were 93.8% (CI 79.2–99.2) and 75.9% (CI 73.1–78.5) for f-Hb ≥4 μg/g and 90.6% (CI 75.0–98.0) and 83.1% (CI 80.6–85.4) for f-Hb ≥10 μg/g. At a higher (“rule-in”) f-Hb threshold of ≥150 μg/g, the sensitivity and specificity for CRC were 78.1% (CI 60.0–90.7) and 95.9% (CI 94.4–97.0). The prevalence of CRC was 3.1%. At the lower limit of f-Hb detection, 73.7% of symptomatic patients had a negative FIT.

**Conclusion:**

FSP demonstrated that FIT identified both a small group of symptomatic patients with a high risk of undiagnosed CRC for urgent investigation and the majority of symptomatic patients with a very low f-Hb who could avoid colonoscopy. Using FIT in this setting should protect patients from unnecessary colonoscopy, diagnose CRC earlier, and optimise colonoscopy utilisation.

## Introduction

In New Zealand (NZ), colorectal cancer (CRC) is the third most common cancer and the second highest cause of cancer-related death (1). Indigenous Māori and Pacific Island peoples have poorer CRC-related survival compared with other populations (1). To reduce CRC burden, the National Bowel Screening Programme (NBSP) was introduced in 2017 as a two-step CRC screening programme where eligible individuals aged 60–74 are offered a biennial quantitative faecal immunochemical test (FIT) with a positivity threshold set at 40 μg Hb/g faeces (2).

Despite the introduction of the NBSP, the majority of CRC patients in NZ are still diagnosed when suspicious symptoms are investigated (2). Symptoms, however, are poor predictors of CRC diagnosis (3, 4). Despite this, contemporary diagnostic pathways for the assessment of patients presenting with symptoms suggestive of CRC rely excessively on symptoms for triaging. This has resulted in an increasing volume of investigations, often overwhelming healthcare resources without producing the expected benefits in CRC-related outcomes (4, 5).

With the publication of a number of relevant guidelines such as the National Institute for Health and Care Excellence (NICE - DG56), Association of Coloproctology of Great Britain & Ireland, and the British Society of Gastroenterology in recent years, alongside significantly restricted access to endoscopy services during the COVID-19 pandemic, there has been increasing interest in using FIT to detect CRC amongst symptomatic patients (6, 7). The majority of published FIT diagnostic performance data for symptomatic patients are from Europe, and questions remain regarding the transferability of FIT diagnostic performance data between different populations and health systems (8–11).

The NZ FIT for Symptomatic Pilot (FSP) was initiated to determine the feasibility and diagnostic accuracy of using FIT as a triaging tool to assess patients presenting with symptoms suspicious for CRC within the NZ public health system, with the hypothesis that FIT could improve prioritisation for colonoscopy.

## Methods

The reporting of this study conforms with the Standards for Reporting of Diagnostic Accuracy Studies guidelines (12). Ethics approval was granted by the New Zealand Health & Disability Ethics Committee (HDEC) (Ethics reference 2024 EXP 19227).

### Study design

The primary aim of the FSP was to describe the performance of a single quantitative FIT for the diagnosis of CRC amongst symptomatic patients presenting in the NZ public health setting. The secondary objectives were to determine the yield and feasibility of expediting colonoscopy for those returning a very high FIT faecal haemoglobin (f-Hb) result (FIT ≥ 150 μg/g), to describe the diagnostic performance of FIT for serious bowel disease (SBD), and to describe the CRC characteristics with very low f-Hb (<10 μg/g).

A key principle of the FSP was to promote equitable participation for indigenous Māori and Pacific peoples through involvement of relevant groups in the development of the FSP study protocol. This resulted in the inclusion of evidence-based equity-enhancing measures, including initial telephone contact, up to five follow-up telephone calls for Māori and Pacific non-responders (one phone call conducted outside of 9 a.m. to 5 p.m.), and the use of ethnically aligned callers to encourage a high FIT return rate amongst these cohorts (13).

The FSP included patients from two Health New Zealand Districts, Waikato and Waitemata, between July 2022 and January 2024. Consecutive adult patients over 18 years of age, referred with symptoms of suspected CRC, who were triaged by secondary care endoscopists to investigation by colonoscopy, were eligible for inclusion. A wide range of symptoms was accepted, consistent with referral criteria outlined by the Ministry of Health, with scope for triaging endoscopist to exercise clinical judgement in evaluating each referral (14).

Consistent with present clinical practice in NZ, all patients invited to FSP with symptoms suspicious for CRC were offered a colonoscopy regardless of the return or result of FIT. Eligible patients were prospectively identified by local endoscopy unit staff who contacted them by telephone initially and invited patients to participate in the FSP. Patients were sent a FIT kit by courier. This contained a specimen collection device, an invitation letter, and test instructions. A prepaid first-class return envelope was provided for patients to post their sample directly to a single, centralised, accredited laboratory for analysis. Approval from HDEC was granted for patients to give implied consent to participate upon returning a FIT sample.

Non-responders were followed up by three phone calls or as above for Māori and Pacific patients. One further FIT kit was sent to non-responders if not received by the patient or if a spoilt FIT result was initially returned.

FSP was facilitated by existing mechanisms of the NBSP. The index test used was a quantitative FIT (OC-sensor, Eiken Chemical Co, Tokyo, Japan). FIT specimen handling, analysis, and quality control were conducted and reported in line with the guidelines for studies on FIT (**Appendix 1**) (15). FIT samples that were unsuitable for analysis or performed post-colonoscopy were recorded as invalid. Quantitative f-Hb results were prospectively recorded by a central study team and were only available to laboratory staff. Patients were blinded to the FIT result, and all were encouraged to attend colonoscopy. Patients with FIT ≥150 μg/g were reported to the local endoscopy unit with instructions to schedule the colonoscopy within 2 weeks of the FIT result. All other invited patients were offered a colonoscopy date within 8 weeks.

Colonoscopy was chosen as the reference standard—the established gold-standard diagnostic method—for a range of colorectal diseases. Endoscopists were blinded to the quantitative FIT result. Colonoscopy procedures were performed by experienced endoscopists with regular review of key performance indicators. Colonoscopy findings and histopathology were electronically reported according to a standardised template.

Data on symptoms and indications for colonoscopy were extracted by the central study team from endoscopy referral forms and endoscopy reports. Other clinical data extracted included colonoscopy findings, pathology findings, and clinical and pathological CRC staging.

The sample size for FSP prioritised feasibility based on estimates drawn from similar studies, meta-analyses, and local data (10, 16, 17).

### Data analysis

Patients were not included in the final diagnostic accuracy analysis if they did not have both a definitive FIT result and a diagnostic colonoscopy.

Findings at colonoscopy were categorised into specific diagnostic categories in a hierarchy, with CRC ranked highest, followed by high-risk adenoma (HRA), inflammatory bowel disease (IBD), low-risk adenoma (LRA), and other non-malignant diagnoses. SBD included CRC, IBD, and HRA. Further details about other diagnostic categories and specific definitions applied in the study are described in **Appendix 2**.

The primary outcome measures were the sensitivity and specificity of FIT for the detection of CRC at specific f-Hb positivity thresholds. Five f-Hb thresholds were chosen *a priori* for analysis based on literature review: 4 μg/g (limit of detection), 10 μg/g, 20 μg/g, 100 μg/g, and 150 μg/g (4, 6, 9, 10, 18). For reference, the positivity threshold for NBSP at the commencement of this study was 40 μg Hb/g faeces (2).

Relevant diagnostic performance measures, including positive predictive value, negative predictive value, positive likelihood ratios, negative likelihood ratios, and the number needed to scope (NNS, i.e., the number of individuals required to undergo colonoscopy to detect one CRC), were similarly reported for each f-Hb positivity threshold (16, 19).

For the description of categorical variables, frequency tables and percentages were used. For the description of continuous variables, mean and medians were used. Missing values were excluded from analyses comparing between groups. Wilson’s method for the binomial distribution was used to calculate 95% CIs. Differences in proportions between categorical groups were evaluated for statistical significance using the *χ* ^2^ test or Fisher’s exact test. *P*-values <0.05 were considered statistically significant. Receiver operating characteristic (ROC) curves were plotted for f-Hb and the diagnosis of CRC.

Analysis was conducted in R software V.4.2.2 (R Core Team, Vienna, Austria) packages epiR and ROCit.

## Results

Some 1,413 eligible patients were identified and sent a FIT kit during the time period. Ultimately, 1,158 (82.0%) returned a definitive FIT result and 255 (18.0%) did not. Of the 20 patients (1.4%) who initially returned spoilt FIT results, nine subsequently returned definitive FIT results after a repeat kit was sent per protocol. Participation rates were significantly lower for patients aged <50 years (73%), male (79%), Māori (73%), and Pacific (72%) (**Appendix 3**).

**Figure 1:**
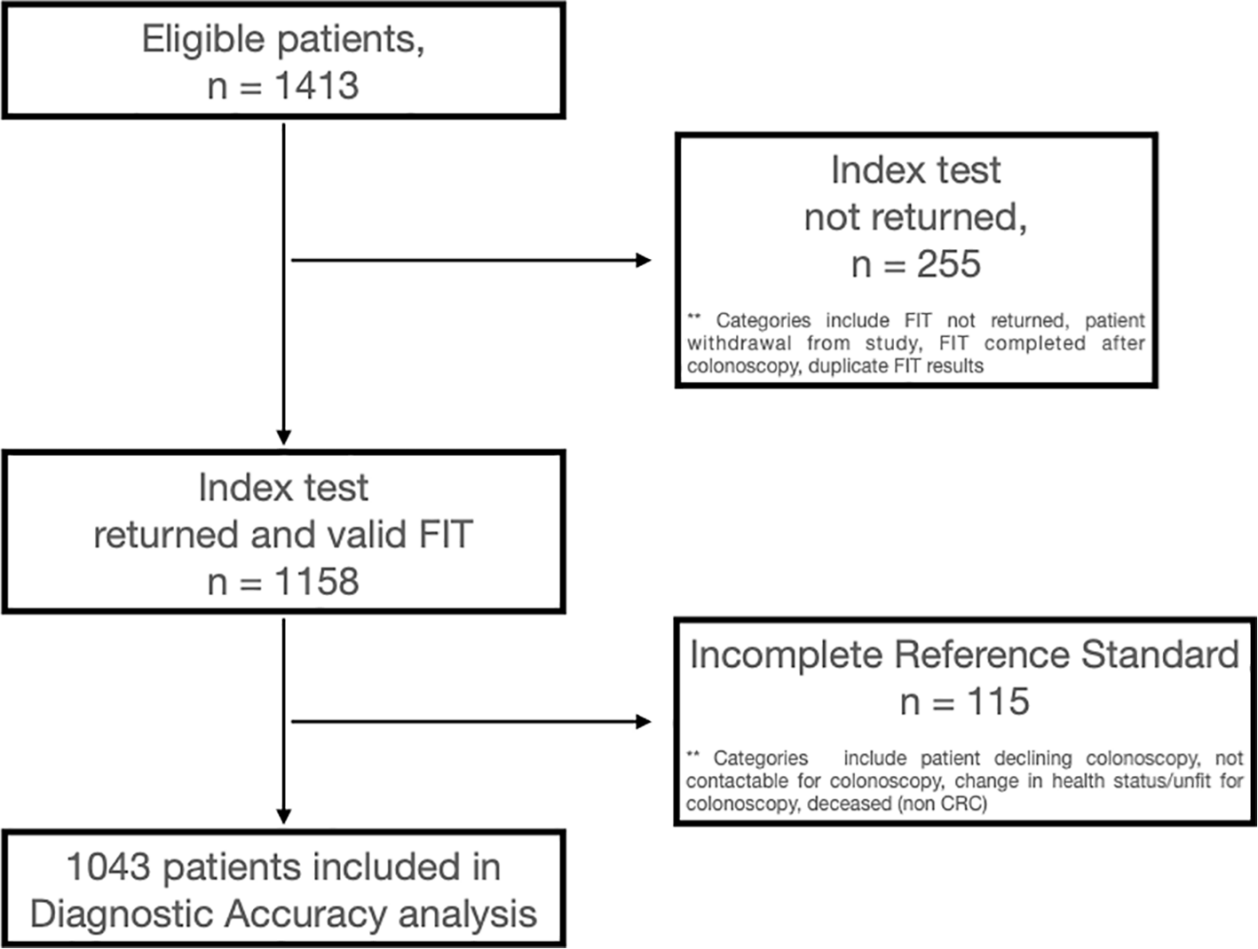
Study flow diagram.

Definitive FIT and colonoscopy results were available for 1,043 patients who were included in the diagnostic accuracy analysis. A study flow diagram is shown in **Figure 1**. Patient demographics of these 1,043 patients who were included in the final diagnostic accuracy analysis are summarised in **Table 1**. The median patient age was 61 years (range: 18–94), and 582 (56%) were women. Ethnicity distribution in the cohort was broadly reflective of the regional population (**Table 1**).

**Table 1:** Patient characteristics included in the diagnostic accuracy study.

	*N*	%
Total	1,043	100.0%
Male	461	44.2%
Female	582	55.8%
Age (years)		
Mean	59.4	
Median (range)	61 (18–94)	
Age group (years)		
<40	115	11.0%
40–59	377	36.1%
60–74	358	34.3%
≥75	193	18.5%
Ethnicity		
Other (NZ European, MELAA)	705	67.6%
Maori	164	15.7%
Asian	122	11.7%
Pacific Islander	52	5.0%
Index of deprivation (NZDEP)		
NZDEP 1–2 (least deprived)	148	14.2%
NZDEP 3–4	218	20.9%
NZDEP 5–6	202	19.4%
NZDEP 7–8	243	23.3%
NZDEP 9–10 (most deprived)	232	22.2%

There were only 32 patients (3.1%) with CRC and 22 (2.1%) with IBD in the cohort (**Appendix 4**). The three most common findings were normal colonoscopy, LRA, and diverticular disease, which together represented 71.9% of colonoscopy findings in this cohort (**Appendix 4**). Of note, 22.3% of colonoscopies in this cohort of symptomatic patients were reported as completely normal, and 1.3% patients in the cohort had readmissions to the hospital within 30 days for various colonoscopy-related complications.

The diagnostic accuracy of FIT for CRC at low (“rule-out”) f-Hb thresholds of 4 μg/g, 10 μg/g, and 20 μg/g is reported in **Table 2**. The proportion of patients whose FIT level exceeded these thresholds was 26.3%, 19.2%, and 15%, respectively. The sensitivity, specificity, and NNS for CRC were 93.8%, 75.9%, and 9.1 for f-Hb ≥4 μg/g; 90.6%, 83.1%, and 6.9 for f-Hb ≥10 μg/g; and 90.6%, 87.4%, and 5.4 for f-Hb ≥20 μg/g. There is a 40-fold increase in NNS to detect one case of CRC when comparing FIT-negative to FIT-positive groups at the f-Hb threshold of 10 μg/g (**Appendix 6**).

**Table 2:** Diagnostic accuracy for CRC, IBD, HRA, and SBD at the proposed “rule-out” f-Hb thresholds of 4 μg/g, 10 μg/g, and 20 μg/g.

F-hb	Test positivity (%)	Most significant diagnostic category	Sensitivity	Specificity	Negative predictive value	Negative likelihood ratio (target < 0.1)	Number needed to scope (NNS) to detect 1 CRC
≥4 μg/g	26.3%	CRC	93.8% (79.2–99.2)	75.9% (73.1–78.5)	99.7% (99.0–99.9)	0.08 (0.02–0.32)	9.1
		IBD	70.8% (48.9–87.4)	59.1% (56.0–62.1)	98.9% (97.9–99.4)	0.49 (0.26–0.92)	25.5
		CRC + IBD	83.9% (71.7–92.4)	77.0% (74.3–79.6)	98.8% (97.9–99.4)	0.21 (0.11–0.38)	5.8
		SBD	62.4% (54.8–69.5)	81.2% (78.4–83.7)	91.3% (89.6–92.7)	0.46 (0.38–0.56)	2.5
≥10 μg/g	19.2%	CRC	90.6% (75.0–98.0)	83.1% (80.6–85.4)	99.6% (99.0–99.9)	0.11 (0.04–0.33)	6.9
		IBD	62.5% (40.6–81.2)	81.8% (79.3–84.2)	98.9% (98.2–99.4)	0.46 (0.27–0.77)	13.3
		CRC + IBD	78.6% (65.6–88.4)	84.2% (81.7–86.4)	98.6% (97.7–99.1)	0.25 (0.15–0.42)	4.5
		SBD	55.6% (48.0–63.1)	88.3% (86.0–90.4)	90.6% (89.1–92.0)	0.50 (0.43–0.59)	2.0
≥20 μg/g	15.0%	CRC	90.6% (75.0–98.0)	87.4% (85.2–89.4)	99.7% (99.0–99.9)	0.11 (0.04–0.31)	5.4
		IBD	58.3% (36.6–77.9)	86.1% (83.8–88.1)	98.9% (98.2–99.3)	0.48 (0.30–0.78)	11.1
		CRC + IBD	76.8% (63.6–87.0)	88.6% (86.4–90.5)	98.5% (97.7–99.1)	0.26 (0.16–0.42)	3.6
		SBD	51.1% (43.5–58.7)	92.5% (90.5–94.2)	90.2% (88.8–91.5)	0.53 (0.45–0.61)	1.7

Values are reported with 95% confidence intervals in brackets.

CRC, colorectal cancer; IBD, inflammatory bowel disease; SBD, serious bowel disease; f-Hb, faecal haemoglobin.

The diagnostic accuracy of FIT for CRC and the test positivity at the proposed higher (“rule-in”) f-Hb thresholds of 100 μg/g and 150 μg/g are summarised in **Table 3**. The proportion of patients whose FIT level exceeded these thresholds was 7.2% and 6.4%, respectively. The sensitivity, specificity, and NNS for CRC were 81.3%, 95.2%, and 2.9 for f-Hb ≥100 μg/g and 78.1%, 95.9%, and 2.7 for f-Hb ≥150 μg/g. Without stratification by f-Hb, the NNS to detect one CRC for this cohort was 32.6.

**Table 3:** Diagnostic accuracy for CRC, IBD, HRA, and SBD at the proposed “rule-in” f-Hb thresholds of ≥100 μg/g and ≥150 μg/g.

F-hb	Test positivity (%)	Most significant diagnostic category	Sensitivity	Specificity	Positive predictive value	Positive likelihood ratio (target > 10)	Number needed to scope (NNS) to detect 1 CRC
≥100 μg/g	7.2%	CRC	81.3% (63.6–92.8)	95.2% (93.6–96.4)	34.7% (27.8–42.2)	16.8 (12.2–23.1)	2.9
		IBD	33.3% (15.6–55.3)	93.4% (91.7–94.9)	10.7% (6.1–18.0)	5.1 (2.8–9.3)	9.4
		CRC + IBD	60.7% (46.8–73.5)	95.9% (94.4–97.0)	45.3% (36.5–54.5)	14.6 (10.1–21.1)	2.2
		SBD	28.7% (22.1–35.9)	97.2% (95.9–98.2)	68.0% (57.4–77.1)	10.3 (6.5–16.3)	1.5
≥150 μg/g	6.4%	CRC	78.1% (60.0–90.7)	95.9% (94.4–97.0)	37.3% (29.6–45.8)	18.8 (13.3–26.6)	2.7
		IBD	25.0% (9.8–46.7)	94.0% (92.4–95.4)	9.0% (4.5–17.0)	4.2 (2.0–8.7)	11.2
		CRC + IBD	55.4% (41.5–68.7)	96.4% (95.0–97.4)	46.3% (36.7–56.2)	15.2 (10.2–22.6)	2.2
		SBD	26.4% (20.1–33.5)	97.7% (96.5–98.6)	70.2% (58.8–79.5)	11.4 (6.9–18.8)	1.4

Values are reported with 95% confidence intervals in brackets.

CRC, colorectal cancer; IBD, inflammatory bowel disease; SBD, serious bowel disease; f-Hb: faecal haemoglobin.

**Figure 2:**
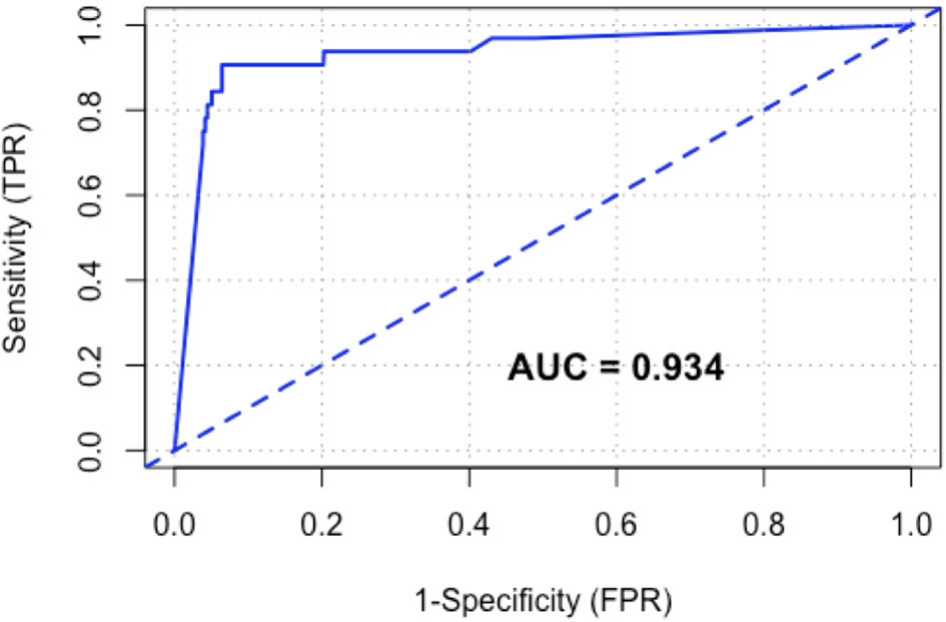
Receiver operating characteristic (ROC) curve of FIT for CRC.

On receiver operating characteristic curve analysis (**Figure 2**), the area under the curve (AUROC) for CRC was 0.93. The point of maximum sensitivity and maximum specificity for this cohort was optimised at 65.6 μg/g.

At low f-Hb thresholds, the diagnostic performance of FIT as a “rule-out (low f-Hb)” test for IBD, HRA, and SBD combined appears inferior when compared to CRC, as summarised in **Table 2**. However, the negative predictive value of SBD at these low f-Hb thresholds remains above 90%. At high f-Hb thresholds, the diagnostic performance of FIT as a rule-in test for IBD, HRA, and combined SBD appears to be retained with very high specificity and positive likelihood ratios when compared to CRC, as summarised in **Table 3**.

Of the 32 CRCs detected in this cohort, 2 recorded f-Hb of <4 μg/g, 5 recorded f-Hb between 4 and 149 μg/g, and 25 recorded f-Hb of ≥150 μg/g.

There were no significant differences between patients with CRC and f-Hb greater or less than either the cutoff of 4 μg/g or 10 μg/g with regard to age, sex, deprivation or ethnicity, anaemia, and iron deficiency anaemia (**Appendix 5**). Interestingly, all three CRCs with f-Hb <10 μg/g were women over 75 years old with proximal CRCs and microsatellite instability.

## Discussion

This is a multicentre, double-blinded diagnostic accuracy study that demonstrated the feasibility of the application of FIT for triaging patients presenting with symptoms suspicious for CRC in NZ. Low negative likelihood ratios, high negative predictive values, and high test sensitivity indicate that FIT at low f-Hb thresholds of 4 μg/g, 10 μg/g, and 20 μg/g is an adequate “rule-out” test for CRC. FIT at high f-Hb thresholds of 100 μg/g and 150 μg/g is an excellent “rule-in” test for CRC, justifying expedited definitive investigation for symptomatic patients with very high f-Hb levels. The majority of symptomatic patients (73.7%) had a negative FIT even at a lower limit of f-Hb detection. A low proportion of patients (6.4%) had a high f-Hb level (≥150 μg/g) but represented 78.1% of all CRCs detected in this study. FIT is a good test for CRC amongst symptomatic patients with an AUROC of 0.93. There is at least a 40-fold increase in NNS to detect one case of CRC when comparing the FIT-negative against FIT-positive groups at low f-Hb thresholds. Taken together, this analysis demonstrated the utility of FIT as an accurate and efficient data-based triaging tool that assists in determining the need for and priority of colonoscopy amongst patients with symptoms suspicious for CRC. False negative FIT at low f-Hb thresholds may be associated with specific tumour characteristics such as primary tumour location and microsatellite instability. Patients who are younger (age < 50), male, and those from ethnic minorities were significantly less likely to participate in a stool test-based programme. FIT is inadequate as a rule-out test for target conditions of IBD and HRA.

Despite high age-standardised incidence rates of CRC in New Zealand, the diagnostic performance of FIT for CRC detection amongst symptomatic patients is comparable to reported diagnostic accuracy measures in the international literature (9, 10, 16). CRC prevalence in this study is also comparable to the reported prevalence (1.1%–7.0%) in published meta-analyses evaluating FIT use amongst symptomatic patients (9, 10). Test sensitivity and specificity for both “rule-in (high)” and “rule-out (low)” f-Hb thresholds reported in this study are remarkably similar to summary estimates of test sensitivity and specificity reported in a meta-analysis involving significantly larger cohort sizes (10). Similar results are noted when the findings of this study are compared directly to the only powered diagnostic accuracy study published on this topic to date (16).

Of the colonoscopies performed in this cohort, 83% had no SBD identified, with the three most common findings being diverticular disease, LRA, and normal colonoscopy. Similar observations have been replicated in other published studies (16, 20). Therefore, in most cases, the findings on colonoscopy do not offer an explanation for the reported symptoms. Colonoscopy use in similar contexts has been identified as an example of low-value healthcare (21). There are other well-established evidence-based and cost-effective uses of colonoscopy resources with demonstrated improvement in CRC outcomes (such as FIT-based population CRC screening) (22). Whilst the importance of increasing colonoscopy capacity to meet increasing demand is undeniable, it is worth noting that the therapeutic capabilities of endoscopy are also expanding. Utilising resource-intensive investigations that are difficult to scale up for low-value healthcare incurs significant opportunity costs (22). Patients and the healthcare system can both benefit by moving to the routine use of simple, more accessible FIT to largely “rule out” CRC for the assessment of symptomatic patients. The reassurance for patients and clinicians that a diagnosis of CRC is unlikely may then allow management to focus on providing symptomatic relief.

Characteristics of missed CRCs or false negative FIT results at very low f-Hb thresholds have been an area of interest in the field despite their low probability. There were no significant differences between patients with CRC and f-Hb greater or less than either low f-Hb threshold of 4 μg/g or 10 μg/g with regard to age, sex, deprivation, ethnicity, tumour stage, or presence of anaemia. However, there appears to be a significant association between false negative FIT at low f-Hb positivity thresholds and proximal location of the primary tumour as well as MSI. These findings must be interpreted with caution due to the small case numbers. Data from larger FIT-based screening cohorts have similarly concluded that false negative FIT results are associated with proximally located CRCs, with smaller studies indicating a possible association with MSI (2, 23, 24). The proposed explanations for this observation include longer colonic transit time leading to greater f-Hb degradation and higher prevalence of non-polypoid tumours or sessile serrated lesions that bleed less in the proximal colon (2). Reliable methods of predicting the presence of proximally located CRC or CRC with MSI will be required to allow practical application of this information to further reduce the probability of false negative FIT at low f-Hb thresholds.

The strengths of this study include a high participation rate with a diagnostic accuracy cohort that is representative of the population where this initiative is planned. Double blinding of the patient and endoscopist, with the utilisation of colonoscopy as the only accepted reference standard, reduced the risk of bias.

However, several limitations need to be acknowledged. In line with the majority of contemporary diagnostic accuracy studies in this area of research, this was not a “powered” diagnostic accuracy study. The low prevalence of CRC in patients referred with symptoms of suspected CRC in NZ, the relatively small population size of NZ, and the high sensitivity of FIT meant that a conventionally powered diagnostic accuracy study was not achievable within a practical timeframe or resources available to the FSP in the wake of the COVID-19 pandemic (10, 16, 17). This study was also conducted at a secondary care level with the possibility of patient selection bias, as not all patients assessed in primary care with these symptoms would have been referred onwards. The wide range of symptoms accepted as an indication for diagnostic colonoscopy and comparatively low CRC prevalence rate reported in this cohort reduced the risk of patient selection bias (10, 17). Although the FSP design did not allow for analysis of time to diagnosis for CRC in this cohort, contemporaneous studies with similar diagnostic pathways have demonstrated a shorter time to diagnosis of CRC for symptomatic patients (25, 26). Whilst overall participation was high, there was a variable participation rate between subgroups of recruited patients (male, age < 50 years, indigenous Māori and Pacific Island patients) despite incorporating evidence-based equity-focused strategies in the pilot. To mitigate the risk of widening existing inequities in CRC care, effective evidence-based strategies that encourage an equitable and high FIT return for all eligible participants are crucial as FIT usage expands.

In conclusion, in the NZ context, FSP has demonstrated that the use of a FIT test with both low and high f-Hb thresholds has the potential to revolutionise the diagnostic pathway of patients with symptoms suspicious for CRC. This could benefit both the patient and the healthcare system. FIT offers the ability to identify a small group (<7%) of patients, representing 80% of the CRCs diagnosed, who can be offered colonoscopy urgently. It also provides evidence suggesting that colonoscopy can be avoided in more than 70% of symptomatic patients because their risk of harbouring CRC is very low (<0.5%). Large real-world evaluations on the implementation of FIT for the assessment of symptomatic patients in NZ with pragmatic safety-netting pathways and cost-effectiveness assessments would help guide the development of evidence-based standards of care. The challenge will be to implement FIT for triaging symptomatic patients in a way that reduces inequities, directs colonoscopy to where it is most urgently required whilst ensuring a diagnostic pathway remains for assessment of patients with persistent symptoms despite a negative FIT.

## Data Availability

The datasets presented in this article are not readily available because requests for de-identified data need to be made through the University of Auckland as data release is subject to approval to ensure respectful use, particularly for indigenous peoples. Requests to access the datasets should be directed to i.bissett@auckland.ac.nz.
