# Supplementary material for: Faecal immunochemical test to triage patients with possible colorectal cancer symptoms: insights from the New Zealand FIT for symptomatic pilot
Source: Front Gastroenterol (Lausanne). 2025 Sep 22;4:1622258. doi: 10.3389/fgstr.2025.1622258 (PMC12952445; doi:10.3389/fgstr.2025.1622258)
Supplement: Supplementary file 1 [file Table1.docx]

**SUPPLEMENTARY MATERIAL**

**APPENDIX 1 - FITTER standards checklist**

**for Labplus Auckland, New Zealand for the New Zealand FIT for Symptomatic Pilot**

*Specimen collection and handling*

Participants were sent a collection device called OC-Auto sampling bottle from Prohealth that provided supplies from Eiken Chemical Co. Ltd. The sample was self-collected by the participants. The green sample probe was removed by twisting and pulling from sampling device. The threaded end of green sample probe was scrapped over the surface of faecal sample until the grooves are filled. After the collection, the probe was placed back into the tube. As the probe passes through the septum into the tube, this allowed the removal of excess faecal material and the optimum amount of faecal material was delivered to the buffer contained within the tube. According to the manufacturer’s specifications there is approximately 10mg of faeces in a 2 ml of buffer. This was transited to lab at room temperature. The sampling tubes were loaded into the analyser racks with the green probe end faced downwards. The OC-Sensor Pledia analyser pierced the foil seal and squeezed the tube to force the buffer liquid through the filter into upper reservoir ready for analysing. Sample analysis was performed as soon as possible according to manufacturer’s specifications. Any sample received later than 14 days of collection was marked as delayed and not processed.

*Analysis*

Specimens were stored at 2-10℃ upon receipt of the samples at the laboratory and processed daily on OC-Sensor Pledia analyser platform. Samples were analysed using latex agglutination immuno-turbidometry method. The analytical working range was 30-1000ng/ml (6-200ug/g). Buffer conversion formula: μg Hb/g faeces = (ngHb/ml buffer)×2mL buffer/10mg faeces collected. Faecal haemoglobin above the upper limit was not diluted and re-analysed.

*Quality management*

Labplus Auckland holds a thorough quality management system and was accredited to ISO 15189 standards by International Accreditation New Zealand (IANZ). All New Zealand FIT for Symptomatic Pilot FIT sample analysis were carried out by professionally accredited medical scientists who were blinded to the results of the reference investigation at time of analysis. The analysers were regularly calibrated according to manufacturer recommendations. For the New Zealand FIT for Symptomatic Pilot, five quality controls were analysed prior to analysis of the samples. These were Level 1 and Level 2 quality control samples provided by the contractor Prohealth. FIT Quality Control was based on an in house quality control reagent which is a dilution of Level 2 control samples. In addition, ASE Low and ASE High quality controls  supplied by Australian Scientific Enterprise were also analysed. In-house inter-batch precision: at 3.4ug/g, CV =19.2%; at 18-93ug/g, CV=1.3-3.9%; at 149ug/g, CV=3.5%

*Data handling*

The New Zealand FIT for Symptomatic Pilot FIT results were recorded manually in an Excel spreadsheet which is password protected. This is inclusive of patient name, national health index number, date of birth, barcode number and the numeric faecal haemoglobin result. A second laboratory medical scientist independently rechecks the information recorded in the spreadsheet against sample readings before information is securely transferred to clinical and research teams.

**APPENDIX 2 - Further details on diagnostic categories and study definitions**

Findings at colonoscopy were categorised into specific diagnostic categories in a hierarchy; CRC ranked highest followed by high-risk adenoma (HRA), inflammatory bowel disease (IBD), low-risk adenoma (LRA) and other non-malignant diagnoses, including diverticular disease, microscopic colitis, benign perianal disease, angiodysplasia , other endoscopic findings and normal colonoscopy for those with no reported abnormalities on the colonoscopy report. Malignant diagnoses other than colorectal adenocarcinomas identified on colonoscopy were recorded as other endoscopic findings. HRA was defined as any polyp with high-grade dysplasia or polyps over 10mm in size with low grade dysplasia, and serrated lesions in the right colon.(1) Other identified polyps less than 10 mm were classified as LRA. Serious bowel disease (SBD) was defined in line with other similar studies to include CRC, IBD and HRA.(1)

CRCs were staged according to the AJCC TNM eighth edition.(2) Primary tumour location was categorised as proximal (caecum to transverse colon) and distal (splenic flexure to rectum). Tumour differentiation grade was categorised as low grade (well-differentiated/grade 1 and moderately differentiated/grade 2) and high grade (poorly/grade 3 and undifferentiated/grade 4). When two or more mismatch protein markers were unstable, the CRC was categorised as a tumour with microsatellite instability (MSI). Patients were classified as anaemic when blood haemoglobin concentration was <120g/L for women or <130g/L for men, based on the most recent measurement within 3months of FIT test date.(3) Iron deficient anaemia (IDA) was present when serum ferritin concentration was <15 μg/L in the context of anaemia.(4) Socioeconomic status was classified based on NZDep2018 Index of Deprivation, an area-based measure of socioeconomic deprivation in New Zealand.(5)

REFERENCES

1. D'Souza N, Georgiou Delisle T, Chen M, Benton S, Abulafi M. Faecal immunochemical test is superior to symptoms in predicting pathology in patients with suspected colorectal cancer symptoms referred on a 2WW pathway: A diagnostic accuracy study. Gut. 2021;70(6):1130-1138.

2. Amin MB, Greene FL, Edge SB, Compton CC, Gershenwald JE, Brookland RK, et al. The Eighth Edition AJCC Cancer Staging Manual: Continuing to build a bridge from a population-based to a more "personalized" approach to cancer staging. CA Cancer J Clin. 2017;67(2):93-99.

3. Organisation. WH. Haemoglobin concentrations for the diagnosis of anaemia and assessment of severity. 2011.

4. Goddard AF, James MW, McIntyre AS, Scott BB, British Society of G. Guidelines for the management of iron deficiency anaemia. Gut. 2011;60(10):1309-1316.

5. Atkinson J, Salmond, C, Crampton, P. NZDep2018 Index of Deprivation, Interim Research Report, December 2019. 2019.

**APPENDIX 3 - Table showing demographic analysis of returned definitive FIT results as indicator of participation in FIT for Symptomatic Pilot**
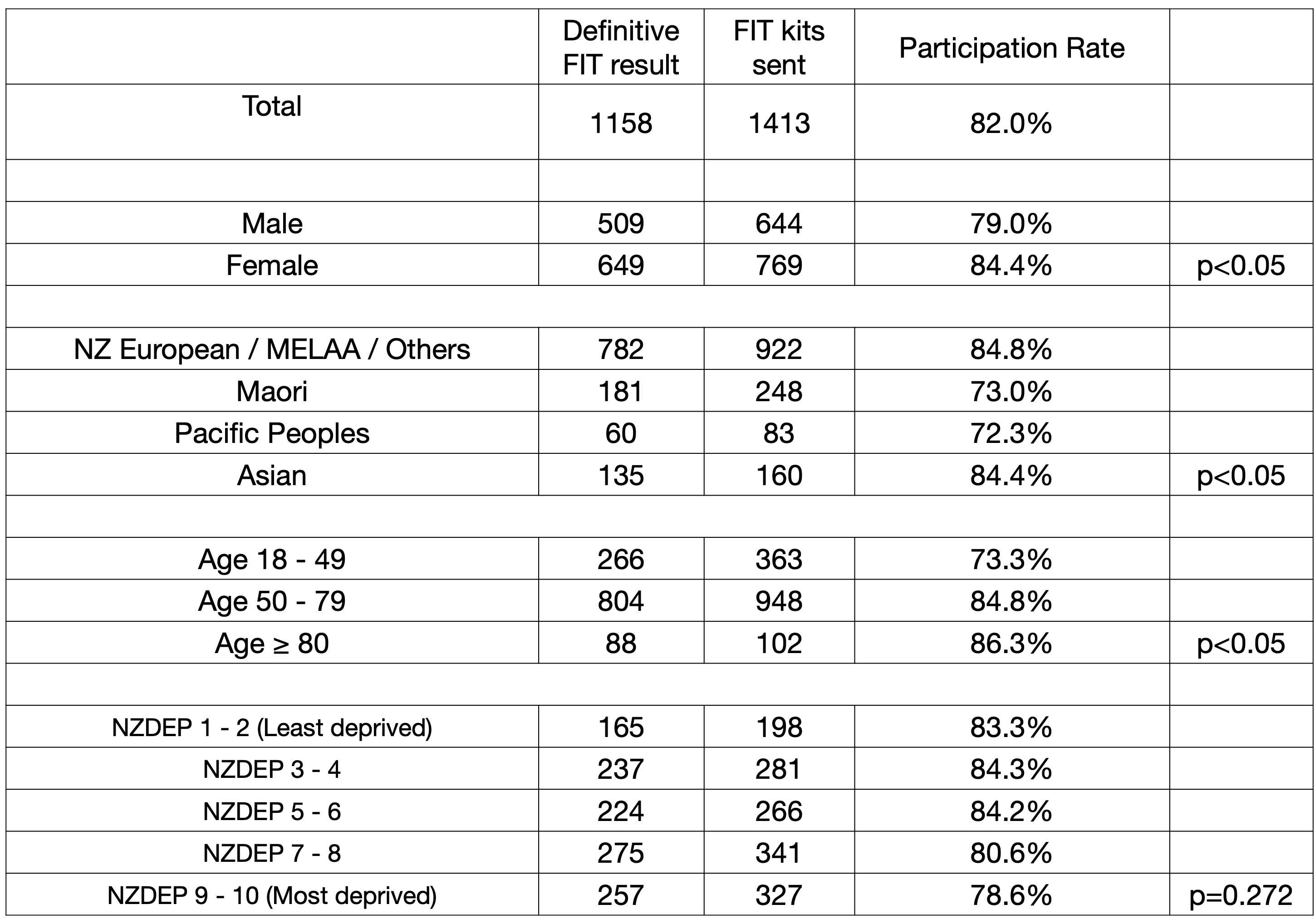


**APPENDIX 4 - Table showing most significant finding / highest ranked diagnostic category for each participant at colonoscopy.**


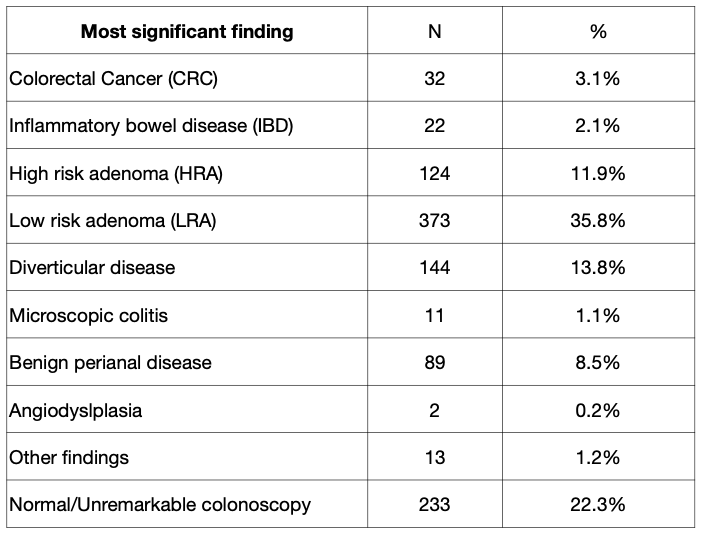


APPENDIX 5 - Table showing characteristics of CRC diagnosed, analysed by false negative FIT results at f-HB of 4 μg/g or 10 μg/g


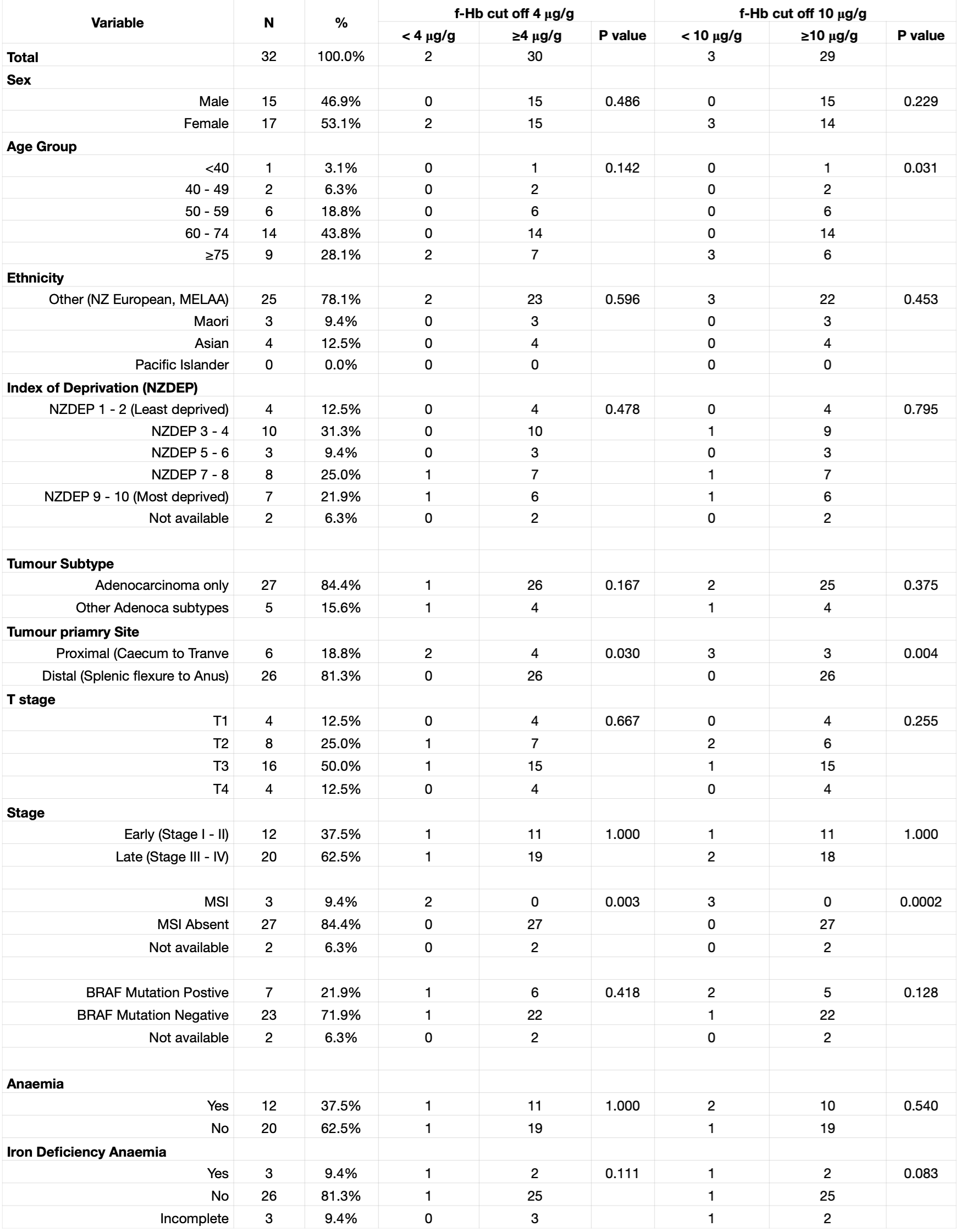


APPENDIX 6 - Diagnostic Accuracy for CRC at proposed “rule-out” f-Hb thresholds of 4 μg/g , 10 μg/g & 20 μg/g

| f-Hb | Test Positivity (%) | Sensitivity | Specificity | NEGATIVE Predictive Value | NEGATIVE Likelihood Ratio  (Target <0.1) | NNS  for  POSITIVE FIT  (Above threshold) | NNS  for  NEGATIVE FIT  (Below threhold) | NNS  Without FIT triaging |
| --- | --- | --- | --- | --- | --- | --- | --- | --- |
| ≥4 μg/g | 26.3% | 93.8%  (79.2 - 99.2) | 75.9% (73.1 - 78.5) | 99.7% (99.0 - 99.9) | 0.08 (0.02 - 0.32) | 9.1 | 384.5 | 32.6 |
|  |  |  |  |  |  |  |  |  |
| ≥10 μg/g | 19.2% | 90.6%  (75.0 - 98.0) | 83.1% (80.6 - 85.4) | 99.6% (99.0 - 99.9) | 0.11 (0.04 - 0.33) | 6.9 | 281.0 | 32.6 |
|  |  |  |  |  |  |  |  |  |
| ≥20 μg/g | 15.0% | 90.6%  (75.0 - 98.0) | 87.4% (85.2 - 89.4) | 99.7% (99.0 - 99.9) | 0.11 (0.04 - 0.31) | 5.4 | 295.7 | 32.6 |

Values are reported with 95% confidence interval in brackets.

CRC: Colorectal cancer; f-Hb: faecal haemoglobin; NNS - Number Needed to scope to detect 1 CRC

APPENDIX 7 - Completed STARD checklist

|  | **Section & Topic** | **No** | **Item** | **Reported on page #** |
| --- | --- | --- | --- | --- |
|  |  |  |  |  |
|  | **TITLE OR ABSTRACT** |  |  |  |
|  |  | **1** | Identification as a study of diagnostic accuracy using at least one measure of accuracy  (such as sensitivity, specificity, predictive values, or AUC) | The manuscript title reflects a study with broader aims as described on page 8. The main analysis performed is a diagnostic accuracy analysis |
|  | **ABSTRACT** |  |  |  |
|  |  | **2** | Structured summary of study design, methods, results, and conclusions  (for specific guidance, see STARD for Abstracts) | #6/20 |
|  | **INTRODUCTION** |  |  |  |
|  |  | **3** | Scientific and clinical background, including the intended use and clinical role of the index test | #7/20 |
|  |  | **4** | Study objectives and hypotheses | #7/20 |
|  | **METHODS** |  |  |  |
|  | *Study design* | **5** | Whether data collection was planned before the index test and reference standard  were performed (prospective study) or after (retrospective study) | #8/20 |
|  | *Participants* | **6** | Eligibility criteria | #8/20 |
|  |  | **7** | On what basis potentially eligible participants were identified  (such as symptoms, results from previous tests, inclusion in registry) | #8/20 |
|  |  | **8** | Where and when potentially eligible participants were identified (setting, location and dates) | #8/20 |
|  |  | **9** | Whether participants formed a consecutive, random or convenience series | #8/20 |
|  | *Test methods* | **10a** | Index test, in sufficient detail to allow replication | #8/20, Supplementary |
|  |  | **10b** | Reference standard, in sufficient detail to allow replication | #9/20 |
|  |  | **11** | Rationale for choosing the reference standard (if alternatives exist) | #9/20 |
|  |  | **12a** | Definition of and rationale for test positivity cut-offs or result categories  of the index test, distinguishing pre-specified from exploratory | #9/20 |
|  |  | **12b** | Definition of and rationale for test positivity cut-offs or result categories  of the reference standard, distinguishing pre-specified from exploratory | #9/20 |
|  |  | **13a** | Whether clinical information and reference standard results were available  to the performers/readers of the index test | #9/20 |
|  |  | **13b** | Whether clinical information and index test results were available  to the assessors of the reference standard | #9/20 |
|  | *Analysis* | **14** | Methods for estimating or comparing measures of diagnostic accuracy | #9/20 |
|  |  | **15** | How indeterminate index test or reference standard results were handled | #9/20 |
|  |  | **16** | How missing data on the index test and reference standard were handled | #9/20 |
|  |  | **17** | Any analyses of variability in diagnostic accuracy, distinguishing pre-specified from exploratory | #9/20 |
|  |  | **18** | Intended sample size and how it was determined | #13/20 |
|  | **RESULTS** |  |  |  |
|  | *Participants* | **19** | Flow of participants, using a diagram | Figure 1. #19/20 |
|  |  | **20** | Baseline demographic and clinical characteristics of participants | Table 1. #15/20 |
|  |  | **21a** | Distribution of severity of disease in those with the target condition | Appendix 4 |
|  |  | **21b** | Distribution of alternative diagnoses in those without the target condition | Appendix 4 |
|  |  | **22** | Time interval and any clinical interventions between index test and reference standard | N/A |
|  | *Test results* | **23** | Cross tabulation of the index test results (or their distribution)  by the results of the reference standard | Table 2. #16/20 |
|  |  | **24** | Estimates of diagnostic accuracy and their precision (such as 95% confidence intervals) | Table 2. #16/20 |
|  |  | **25** | Any adverse events from performing the index test or the reference standard | #10/20 |
|  | **DISCUSSION** |  |  |  |
|  |  | **26** | Study limitations, including sources of potential bias, statistical uncertainty, and generalisability | #12 - 13 / 20 |
|  |  | **27** | Implications for practice, including the intended use and clinical role of the index test | #13/20 |
|  | **OTHER INFORMATION** |  |  |  |
|  |  | **28** | Registration number and name of registry | #8/20 |
|  |  | **29** | Where the full study protocol can be accessed | N/A |
|  |  | **30** | Sources of funding and other support; role of funders | #4/20 |
|  |  |  |  |  |
